# Methods Training to Advance Integrative Science on Minority Aging

**DOI:** 10.1093/geroni/igab046.1177

**Published:** 2021-12-17

**Authors:** Briana Mezuk, Monica Firestone, Wassim Tarraf

**Affiliations:** 1 University of Michigan, Ann Arbor, Michigan, United States; 2 Department of Epidemiology, University of Michigan School of Public Health, Michigan, United States; 3 Institute of Gerontology & Department of Healthcare Sciences, Wayne State University, Detroit, Michigan, United States

## Abstract

Minority aging is an inherently interdisciplinary field. However, it can be difficult for early-career investigators to develop skills on how to integrate data sources, study designs, measurement approaches, and analytic tools from disparate fields into their research programs. This session will illustrate how the biopsychosocial framework has been used to structure the content and delivery of methods training related to minority health/aging research in two NIH-funded exemplar programs: the MCUAAAR Analysis Core, and the Michigan Integrative Well-Being and Inequality (MIWI) Training Program. This talk will illustrate how the 20-year history of MCUAAAR informed the development of MIWI, and how both initiatives approach early-career scientist training through: i) centering learning within a mentorship structure to model team science, ii) avoiding false dichotomizes and hierarchies in study designs and data sources, and iii) attending to the unique challenges faced by scientists working in minority health through knowledge sharing.

